# Length of intraabdominal measurement of bowel (LIMB)

**DOI:** 10.1016/j.sopen.2023.09.018

**Published:** 2023-09-21

**Authors:** Danielle Patrick, Kayla Rizzo, Sam Grasso, John Schriver

**Affiliations:** William Beaumont Army Medical Center, 18511 Highlander Medics Street, El Paso, TX 79918, United States of America

**Keywords:** Bariatric surgery, Gastric bypass, Residency, Graduate medical education, Laparoscopy

## Abstract

**Background:**

Laparoscopic Roux-en-Y gastric bypass (RYGB) is one of the most commonly performed bariatric surgeries. The steep associated learning curve is dependent on the training facility, laparoscopic experience, and overall procedural volume. William Beaumont Army Medical Center (WBAMC) has been accredited as a bariatric center of excellence and trains resident surgeons in the performance of RYGB.

**Objective:**

This study aimed to investigate the accuracy and precision of a bariatric center of excellence's training of surgical residents in terms of laparoscopic measurements of simulated small bowel. This will act as a surrogate for how well surgical residents learn to run the small bowel during bariatric procedures and how their accuracy and precision change over time in training.

**Setting:**

This study took place at William Beaumont Army Medical Center, a bariatric center of excellence and training institution.

**Methods:**

Participants included surgical residents from WBAMC. Participants used a laparoscopic trainer and two bowel graspers to measure both a collapsing garden hose (simulated bowel) and a nylon rope (control material) to 75 cm (cm) and 125 cm (cm), three times each, with recordings of time required to do so, actual distance measured, and technique used.

**Results:**

Fifteen residents participated in the study. Residents displayed accuracy of 21.6 %. 33%of residents were precise for the 75 cm measurement, and 53 % of residents were precise for the 125-cm measurement. PGY-4 residents were the most accurate while PGY-3 residents were the most precise. There were no statistical differences between junior (PGY 1–4) and senior residents (PGY 5–6) in accuracy or precision in the measurement of 75-cm or 125-cm. No statistical differences were found measuring the hose versus rope in accuracy nor precision. PGY-4 residents completed the task in the least amount of time while PGY-2 residents took the longest to complete each task.

**Conclusions:**

In general, residents are neither precise nor accurate in measurements of simulated bowel lengths, and experience does not contribute to either. Time in residency correlates with laparoscopic speed but not with accuracy nor precision. Extrapolating this data to attending surgeons suggests that estimated lengths of small bowel that are ‘run’ or measured during laparoscopic cases are neither accurate nor precise. More investigation must be performed in this area.

## Highlights

• Residents are neither precise nor accurate at measuring simulated bowel lengths
• Junior residents are equally precise and accurate as senior residents in simulation
• Time in residency correlates to laparoscopic speed

## Introduction

America continues to experience an obesity epidemic. This is also occurring worldwide. Accordingly, the number of bariatric procedures continues to increase annually, with an estimated 256,000 procedures performed in 2019, up from 158,000 in 2011 [1], and the majority of procedures are performed by formally trained minimally invasive and bariatric surgeons. The most common bariatric surgery performed is laparoscopic sleeve gastrectomy (LSG) followed by laparoscopic Roux-en-Y gastric bypass (RYGB) [1]. For the training of general surgeons, the Accreditation Council for Graduate Medical Education provides defined category minimum numbers for residents to complete in order to successfully graduate [2]. General surgery residents must complete at least 40 stomach and small intestine cases (which would include LSG and RYGB). They also must complete 75 complex laparoscopic cases, which also include the aforementioned bariatric procedures. Graduates of a general surgery residency program should be competent and confident in the performance bariatric surgery in their practice when indicated, as the American Board of Surgery indicates expectations of such knowledge upon certification [3]. It should be noted that though general surgeons may not be performing bariatric operations regularly or at all, intracorporeal measurement of both small and large bowel can be of importance in certain operations, such as cases of bowel resection for malignancy where adequate margins are critical.

A systematic review published in 2020 [4] aimed to define the learning curves for LSG and RYGB and found significant heterogeneity between studies, with variations depending mostly on patient outcomes and surgical efficiency, level of experience with laparoscopy, and case volume. The learning curve for RYGB was found to be between 30 and 500 procedures; more specifically between 30 and 50 procedures to be determined a competent surgeon. In this case, a competent surgeon was one who follows guidelines, plans and carries out an intended operative plan, and is safe.

A key component in the conduct of a RYGB operation involves measuring the small bowel at certain distances, previously described anywhere between 30 and 50 cm and 125–200 cm, depending on surgeon preference and operative technique. Despite the necessity of performing this “running of the small bowel” maneuver, in bariatric surgery and other types of surgery, to date there is no standard method for doing so. There is also no standardized means for intracorporal measurement of small bowel length. Various techniques may be used and are dependent on surgeon preference and training. These include, but are not limited to, visual estimations, using laparoscopic instrument markings for a more guided visual estimate, or by means a ruler or other pre-measured length of material (umbilical tape, suture, etc.). A recent study found that the use of pre-measured tape was more accurate than visual judgement or by utilizing markings on a laparoscopic instrument (i.e., bowel graspers). Of note, when using this technique, participants underestimated bowel length [5]. It is likely, then, that there is relative variability in perceived bowel length measurements between surgeons, and perhaps even within a surgeon's own abilities. This variability and source of error can be further compounded by having to train residents that are trying to learn how to perform the procedures.

Weight loss outcomes may be affected by varying biliopancreatic (BP) or alimentary limb lengths, where Negaard et al. [6] found that patients with long BP limbs (2 m) had increased weight lost compared with short BP limbs (60-cm) at both short- and long-term intervals. However, this same study also found that patients with longer BP limbs experienced more gastrointestinal symptoms, malabsorption, and increased need for vitamin supplementation [6]. Outcomes regarding varying lengths of the roux and BP limbs and the common channel have been a discussion of debate, though the recently published mid-term analysis of Dutch Common Channel Trial (DUCATI) [7] found, at 3-year follow up, a significant decrease in percent total weight loss, percent excess weight loss, and resolution of type 2 diabetes mellitus in patients who underwent RYGB with a very long roux limb (60-cm BP limb, 100-cm common channel, variable roux limb) compared with patients who underwent RYGB with a standard roux limb (60 cm BP limb, 150 cm roux limb, variable common channel). Thus, variability of perceived bowel length measurements may affect patient outcomes clinically.

The rate of obesity within the United States Department of Defense (DoD) has remained fairly stable at 17 % between 2019 and 2020 [8]. Hundreds of bariatric procedures are performed within the DoD yearly, and William Beaumont Army Medical Center (WBAMC) is known as a Bariatric Center of Excellence. WBAMC hosts a general surgery residency program in which bariatric surgery is emphasized. The purpose of this research project is to investigate the accuracy and precision of resident measurements of simulated small bowel in a simulated laparoscopic environment. Data collection will include level of training, technique used, accuracy and precision of measurements. We hypothesize that residents in their final chief year will have the most accuracy and least variability when compared with their junior counterparts. Additionally, we hypothesize that residents in their final chief year will perform these tasks the quickest.

## Materials and methods

After presentation to our international review board, a formal ethics approval was found to be unnecessary as patients were not involved. Participation was voluntary, and verbal informed consent was obtained from each participant. Potential subjects included all current residents in the general surgery residency program at WBAMC, including residents in the research (i.e., non-clinical) year. Participants were recruited by means of announcements at general surgery residency program events, such as morning conference. There were no exclusion criteria.

A standard laparoscopy trainer box with a fixed, mounted camera was used. Images were displayed on a monitor, and participants were provided with two bariatric-length laparoscopic bowel graspers. All participants used the same trainer box and graspers. A stretchable, collapsible garden hose and a nylon rope, each measuring 250-cm, were used in all experiments so as to simulate small intestine reproducibly. The garden hose was measured first and was secured at one end using a clip within the trainer box. Participants were asked to measure the hose starting at the clip to a length they thought to be 75-cm, and to clamp down on the hose at that distance. Participants were instructed and permitted to use any desired method to achieve the target measurement. The hose was measured from the starting point to the clamped grasper by a member of the research team. The participants were blinded to their measurements between repetitions. No feedback was disclosed to the participant during their performance, nor after each measurement was taken. This process was repeated for a total of three trials of the 75-cm measurement, and the hose and trainer box were reset between each trial. This process was again repeated with the hose for a targeted length of 125-cm, again three times with materials reset between each trial. After completion of all trials with the hose, these same steps were repeated with the nylon rope. Each trial was timed, and technique for measurement was also noted.

### Statistical methods

Student *t*-test was utilized to compare trends between junior and senior residents as well as the whole cohort for both measurements and time. Relative standard deviations were applied to assess precision and accuracy. Precision was defined as within 10 % of the relative standard deviation (RSD). Accuracy was defined to be within 10 % of the true value. A standard *p* value <0.05 determined statistical significance.

## Results

Fifteen residents from the general surgery residency program at WBAMC participated in the study. This included: four post-graduate year (PGY) 1 interns, three PGY2 (research residents), two PGY3, two PGY4, two PGY5, and two PGY6 (chief residents). Ninety-three percent of participants (14/15) visually estimated measurements, whereas one participant created a pre-measured strip of paper and inserted it into the trainer box.

Overall, residents displayed accuracy of 21.6 %. Residents exhibited precision 33 % of the time for the 75-cm length and 53 % of the time for the 125-cm length. Again, precision was defined as within 10 % of the RSD, whereas we defined accuracy to be within 10 % of the true value.

**Fig. 1:**
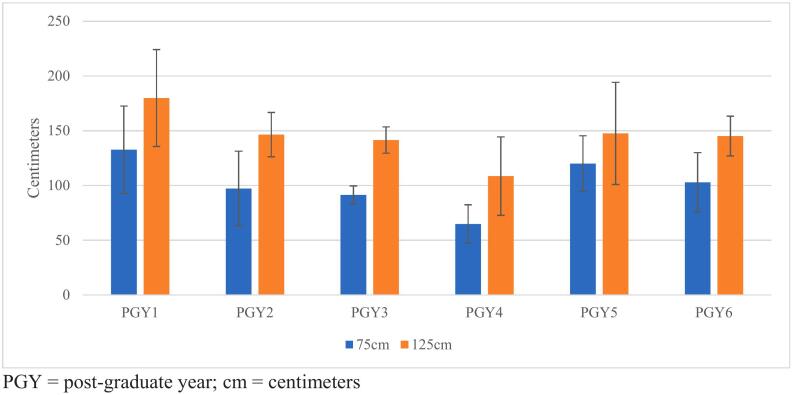
Overall measurements with standard deviation, by PGY year PGY = post-graduate year; cm = centimeters.

Fig. 1 displays measurements for both 75-cm and 125-cm trials overall, broken down by PGY year. PGY4 residents were the most accurate for both 75-cm and 125-cm measurements, whereas PGY1 interns were the least accurate (mean 64.9 cm and 108.5 cm for PGY4 vs. mean 132.7 cm and 179.8 cm for PGY1 for 75-cm and 125-cm lengths, respectively). Additionally, PGY3 residents were the most precise whereas PGY1 interns were the least precise.

Junior residents (PGY1-PGY4 years) were then compared to senior residents (PGY5-PGY6 years). For the 75-cm measurement, the average measurement was 101.8 cm and 111.4 cm for junior and senior residents, respectively (*p* = 0.38, Table 1). Similarly for the 125-cm measurement, junior residents measured an average 143.6 cm whereas senior residents measured on average 146.3 cm (*p* = 0.80, Table 1). It should also be noted that there was no statistical difference in measurements using either the stretchable garden hose or the nylon rope as a medium for both 75-cm and 125-cm measurements (*p* = 0.50 and *p* = 0.18, respectively).

**Table 1:** Comparing junior residents (PGY1-PGY4) versus senior residents (PGY5-PGY6) between 75 cm and 125 cm overall measurements.

Length	PGY	Mean (cm)	SD	*P*-value
75 cm	1–4	101.8	39.7	0.38
	5–6	111.4	28.5	
125 cm	1–4	143.4	41.5	0.80
	5–6	146.3	36.6	

SD = standard deviation.

**Fig. 2:**
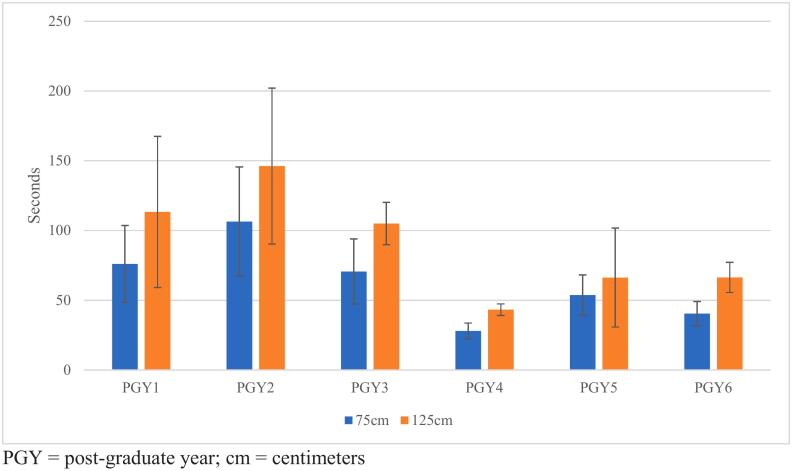
Time in seconds, by PGY year PGY = post-graduate year; cm = centimeters.

Average trial times, broken down by PGY subgroup, are displayed in Fig. 2. PGY4 residents completed the trials in the least amount of time (28 s and 43.25 s for 75 cm and 125 cm lengths, respectively), while PGY2 residents, residents on their research year, required the most time (106 s and 145 s, for 75 cm and 125 cm lengths respectively).

Technique for measurement was also noted during data-gathering. The most common method was ‘hand-over-hand.’ Some residents counted out loud while others counted silently to themselves. Additionally, few residents ran out of hose or rope length prior to completing the requirement trial length, indicating their technique for measurement was largely overestimating.

## Discussion

The purpose of the current study was to assess the accuracy and precision of residents in a general surgery program in measuring simulated small bowel lengths. We predicted that residents in their chief years (i.e., more experienced residents) would be both more precise and accurate, as well as complete the tasks in the least amount of time. At least two key findings are within the present analysis. First, overall, general surgery residents are neither precise nor accurate in measurements of simulated bowel lengths, and experience does not contribute to either. Second, time in residency does not correlate with speed. We could extrapolate to suggest general surgery residents at this institution do not following a particular learning curve in regards to accuracy, precision, or timing in the conduct of running lengths of small bowel, which has clinical implications for performing bariatric surgery.

To date, there are no known studies in which residents from a general surgery program are observed to measure simulated bowel in a laparoscopy trainer box. This residency program does not utilize a formal laparoscopy simulation training curriculum; however, this tool is openly available for residents to use for practice at any time. There are other simulation tools available to these residents, including a computerized laparoscopic simulator.

Based on data displayed in Fig. 1, PGY1 interns are both the least accurate and least precise. This is not surprising given their minimal operative experience, and even more so their substantially less laparoscopic experience. PGY4 residents were the most accurate, and PGY3 residents were the most precise, even when compared to senior/chief residents; accuracy and precision do not correlate with time in residency. These findings are likely to correlate with the systematic review by Wehrtmann et al. [4], which found the learning curve for RYGB to be between 30 and 500 cases; though it is uncertain how many bariatric cases, specifically RYGB, each resident had performed at the time of the present study. This study also found that the learning curve was easier to overcome at institutions with higher case volume and in surgeons with increased laparoscopic experience [4]. More likely, chief residents have performed more RYGB cases than their junior counterparts, however given the increased popularity of LSG over RYGB, that may contribute to the overall poor accuracy and precision. Additionally, with the recent Coronavirus-19 (COVID) pandemic, elective surgeries including bariatric surgeries were cancelled for a period of time at our institution; overall operative volume was certainly compromised.

Time in residency does not correlate with speed for measuring simulated bowel, as evidenced in Fig. 2. This is contradictory to what would be intuitive; efficiency, and specifically speed, should increase as more cases are performed and techniques are mastered. However, the PGY4 residents completed the tasks in the shortest amount of time. It is not surprising here that the PGY2 residents took the longest to complete the tasks, as these residents are in their research (i.e., non-clinical) year and have very minimal clinical exposure, and any skill obtained during intern year may have diminished. This study suggests that personal surgical practice and technique may affect overall surgical speed, rather than time in training.

This study demonstrates that experience, and seemingly more operations involving bowel being performed, during residency does not correlate to accurate or precise measurements of bowel. This could indicate the need for formalized laparoscopy training within a residency program, rather than relying on the number of case performances. This especially may be helpful for those residents whose education may have been compromised by the recent COVID pandemic, with cancellation of multiple elective (i.e., bariatric) cases. However, given the inconsistent nature of advanced laparoscopic surgery rotations within a residency program, a longitudinal cohort study may demonstrate relatively different outcomes compared to the current study. This could be an area of future research.

There are limitations to this study. The study is underpowered, and its sample size is certainly inadequate; however, this study took place at a single institution and within a single residency program. Future studies may include a larger sample size (i.e., more residency programs in the local community) or following residents longitudinally to see how each resident may progress. Additional studies may focus on actual laparoscopic RYGB cases, again following residents over time throughout their course of training. Another potential limitation is the material utilized for bowel simulation. A flexible garden hose, while it does have some elastic properties, may not be the best simulation material for use in this study.

This raises important questions – when does a surgeon become competent and/or consistently perform accurate and precise measurements of length of bowel? Are small bowel lengths in RYGB actually accurate to the reported numbers? Does a singular surgeon have significant variability between their roux limb lengths? This is an area for future studies, perhaps within a minimally invasive and bariatric surgical fellowship program.

The number of bariatric surgeries performed each year continue to rise in light of the ongoing and escalating obesity epidemic. Residents graduating from general surgery residency should be competent in the conduct of bariatric surgeries including LSG and RYGB. The current methods for educating patients on estimating small bowel lengths, as done in RYGB, are lacking and are neither accurately nor precisely being performed.

## Conclusions

General surgery residents in training are neither precise nor accurate in measurements of simulated bowel lengths, and experience does not contribute to either. Time in residency correlates with laparoscopic speed.

## Disclosures

The authors of this manuscript have no disclosures. “The views expressed in this publication are those of the author(s) and do not reflect the official policy or position of William Beaumont Army Medical Center, Department of the Army, Defense Health Agency, or the US Government.”

## CRediT authorship contribution statement

Danielle Patrick: Data collection, original draft preparation

Kayla Rizzo: Original concept, draft revisions

Samuel Grasso: Original concept, data collection, draft revisions

John Schriver: Supervision

## Ethical Publication

This study was approved by our institution's IRB.

## Declaration of competing interest

There are no conflicts of interest. This project was not funded by any source.

## Abbreviations

RYGB: laparoscopic roux-en-y gastric bypass
WBAMC: William Beaumont Army Medical Center
cm: centimeter
LSG: laparoscopic sleeve gastrectomy
BP: biliopancreatic
DUCATI: Dutch Common Channel Trial
DoD: Department of Defense
RSD: relative standard deviation
PGY: post-graduate year (from medical school)
COVID: Coronavirus-19
